# Transcriptome Analysis of *Ophraella communa* Male Reproductive Tract in Indirect Response to Elevated CO_2_ and Heat Wave

**DOI:** 10.3389/fphys.2020.00417

**Published:** 2020-05-05

**Authors:** Xuyuan Gao, Zhenya Tian, Yan Zhang, Guangmei Chen, Chao Ma, Zhenqi Tian, Shaowei Cui, Yongyue Lu, Zhongshi Zhou

**Affiliations:** ^1^College of Agriculture, South China Agricultural University, Guangzhou, China; ^2^State Key Laboratory for Biology of Plant Diseases and Insect Pests, Institute of Plant Protection, Chinese Academy of Agricultural Sciences, Beijing, China; ^3^Guangxi Key Laboratory of Biology for Crop Diseases and Insect Pests, Institute of Plant Protection, Guangxi Academy of Agricultural Sciences, Nanning, China

**Keywords:** *Ophraella communa* LeSage, carbon dioxide, heat wave, transcriptome, seminal fluid proteins

## Abstract

Increase in atmospheric CO_2_ directly affects the insect physiology and behavior, and indirectly affects the herbivorous insects by affecting their hosts. The increase in atmospheric CO_2_ is accompanied by an increase in temperature and heat waves. *Ophraella communa* LeSage is a natural enemy of *Ambrosia artemisiifolia* (common ragweed). The development and reproduction of this beetle is weakened upon eating common ragweed grown under stress conditions. As female behavior and physiology alter after mating, the reproductive tract of males is likely to modulate reproduction and development in this species. Herein, the transcriptional profiles of testes and accessory glands from male *O. communa* individuals feeding on common ragweed under conditions of high CO_2_ concentration and heat waves and that grown under ambient CO_2_ concentration were compared. Differentially expressed genes (DEGs) were identified between the same tissues from beetles fed on common ragweed grown under different stress conditions. There were 3, 2, 3, 1and 5 genes related to decomposition and transport of macromolecular substances, host location, stress response, reproduction, and poisonous food-utilization. No expected response was observed in the male reproductive tract, but some of the identified DEGs might control the development of the population. The results presented here should be helpful in guiding future studies on deciphering the indirect response of other organs to high CO_2_ concentration and heat waves, as well as the functions of seminal fluid proteins in *O. communa*.

## Introduction

Global warming is increasing and has been one of the greatest challenges facing all organisms (Parmesan and Yohe, 2003). According to a special report of the Intergovernmental Panel on Climate Change (IPCC), the global average surface temperature has risen by 1.5°C above that in the pre-industrial era and is expected to keep on increasing for decades or centuries (Bongaarts, 2018). The warming climate has had a significant effect on organisms as well as on the ecosystems (Hughes, 2000; Parmesan and Yohe, 2003; Zvereva and Kozlov, 2006). In the future, extreme weather events are expected to increase further, heat waves (short-term extreme heat), defined as the extreme temperatures, may increase in frequency, severity, duration, or areal extent (Robinson, 2001; Della Marta et al., 2007; Griffiths and Bradley, 2007; Dosio, 2017). Heat waves are extremely high temperatures that can cause damage or death to humans and can damage plants in a short time (usually in a few days) (Hunt, 2007). In recent years, many countries and regions, such as those of Europe and the Americas have been reportedly hit by heat waves (Keellings et al., 2018; Trimmel et al., 2018; Rita et al., 2019). In China, heatwaves are experienced over a vast area (Gao et al., 2015). The greenhouse effect caused by elevated CO_2_ concentrations is one of the most common global warming phenomenon (Guo et al., 2009). As evidenced by data from the Mona Loa Mountain Meteorological Observatory in Hawaii, global atmospheric CO_2_ concentrations increased from 280 ppm before the Industrial Revolution to 401 ppm in 2016 and would further increase to ∼540–970 ppm (average 700 ppm) by the end of the 21st century (Zvereva and Kozlov, 2006). This indicates that heat waves will be particularly prominent in the context of climate warming.

Insects are poikilothermic animals. Their development, reproduction, physiology, and metabolism are significantly affected by global warming (Murray et al., 2013; Couture et al., 2015; Sentis et al., 2017; Ma et al., 2018). Increased CO_2_ concentrations are associated with substantial changes in the photosynthetic activity of plants (especially C_3_ plants), promote their growth and expansion (Tripathee, 2008), and increase the content of secondary defense substances, which indirectly affects the development and reproduction of herbivorous insects (Bidart-Bouzat and Imeh-Nathaniel, 2008; Murray et al., 2013). Heat waves are rare, extremely high temperature events that have a significant effect on plant growth, photosynthesis, physiology, and metabolism in the short term. The increase in CO_2_ concentration is always accompanied by an increase in temperature. Therefore, the comprehensive stress caused by high concentrations of CO_2_ and heat wave may increase the indirect effects on the growth, development, and population fecundity of herbivorous insects, whose phenotypes and genes would change accordingly.

*Ambrosia artemisiifolia* (common ragweed) originated in the Sonoran region of North America and is a C_3_ plant. As a malignant and invasive weed, it poses a serious threat to human health, agricultural production, and biodiversity (Vincent et al., 1992; Fumanal et al., 2005, 2007; Bohren, 2006; Bonini and Ceriotti, 2019). *Ophraella communa* LeSage (Coleoptera: Chrysomelidae) is derived from North America and its larvae and adults feeds on common ragweed leaves. This insect has been used as a natural enemy with good prospects for the control of common ragweed in the United States, Mexico, Canada, Japan, South Korea, and China (Palmer and Goeden, 1991; Sohn et al., 2002; Tamura et al., 2004; Zhou et al., 2010a, b). In China, *O. communa* has been widely used for effective control of common ragweed in Hunan, Hubei, Jiangxi, Guangdong, Guangxi, Fujian, and other provinces (Zhou et al., 2010a). However, we made an interesting observation in a previous study that the hottest days of the summer (colloquially referred to as the “dog days”) adversely effected the survival, development, and fecundity of *O. communa* and the population of this beetle showed an obvious decline. In contrast, the growth of common ragweed was the best during these days. In the same time, Bae et al. (2019) found the plant growth and the concentrations of alleochemicals in common ragweed significantly increased in elevated CO_2_. We have also noticed previously that the contents of secondary defense substances, such as tannins and phenols, in common ragweed leaves are significantly increased under the combined stress imposed by high CO_2_ concentration and heat waves. Further, we found that the fecundity and lifespans of control females (feeding on common ragweed under ambient CO_2_ concentration) were significantly decreased while mated with treated males (feeding on common ragweed under the stress of high CO_2_ concentration combined with the heat wave); the fecundity and lifespans of treated females did not show a clear change while mated with control males (unpublished work). Therefore, it is believed that by feeding on common ragweed growing under the stress imposed by a combination of high CO_2_ concentration and heat waves, the reproductive function of *O. communa* adults is significantly reduced, and the breeding ability of the population is reduced.

Male insects ejaculate sperm into the females during mating. In addition to sperm, a large number of seminal fluid proteins (SFPs) and non-protein small molecules are present in the seminal fluid. SFPs are the main effectors required for postmating physiological and behavioral changes in insect females. These changes include reduction in the likelihood of female re-mating, increase in female egg production, changes in female flight and feeding behavior, and alterations in female gene transcription and female genital structures (Avila et al., 2011). The SFPs are mainly produced in the male accessory glands, and the testes also secrete some of them (Avila et al., 2011). The function of insect SFPs has been studied most intensively in Drosophila (Wolfner, 2002, 2007; Ravi Ram and Wolfner, 2007). Studies on the function of SFPs have also been carried out in mosquitoes (Dottorini et al., 2007; Sirot et al., 2008; Rogers et al., 2009), bees (Collins et al., 2006; Baer et al., 2009), cockroaches (Braswell et al., 2006; Andrés et al., 2008), and red pirates (South et al., 2011). It is hypothesized that feeding on common ragweed grown under high CO_2_ concentration and heat wave stress has an indirect effect on the growth and reproduction of *O. communa* females, and the regulatory function of the SFPs may be an intrinsic reason for this effect. Nevertheless, no studies have been conducted on the SFPs of *O. communa*. Our understanding about the mechanisms underlying this relationship between herbivores and plants under conditions of elevated CO_2_ concentrations and heat waves is also very limited. Thus, the expressions of genes in the accessory glands and the testes (tissues of secreting SFPs) of *O. communa* males were compared. And the males were fed on common ragweed under elevated CO_2_ concentration and heat waves, while the males feeding on common ragweed under ambient CO_2_ concentration were as control. The results described herein would lay a good foundation for exploring the detailed functions of SFPs and might be helpful in explaining the mechanism underlying the indirect effects of CO_2_ and heat wave stress in the decline of the reproductive ability of *O. communa*.

## Materials and Methods

### Insect Collection and Dissection

*Ophraella communa* individuals were given different treatments of common ragweed since the egg stage. The eggs were produced on the same day by the same batch of adults raised in the laboratory. First, common ragweed seeds were sown in plastic nutrient bowls (Φ15 cm) with 3–5 seeds in each. The substrate was a 1:2 mixture of vermiculite: flower nutrient soil. Two groups—experimental and control—were set up. The seeds were germinated and grown in artificial climate chambers that can control temperature, humidity, photoperiod, light intensity, and CO_2_ concentration [Model: PRX-450-CO_2_ type; temperature range (°C): ∼0–65 ± 0.5°C; humidity range (RH): ∼25–95 ± 2%; produced by Shanghai COLIN Experimental Instrument Factory]. For the experiment group, the growth conditions were: 30 ± 1°C day temperature, 25 ± 1°C night temperature, 70 ± 5% relative humidity, 14-h L:10-h D photoperiod, 30,000 lux light intensity and 700 ppm CO_2_ concentration. When common ragweed grew to a height of about 10 cm, two robust seedlings were retained per bowl. As the plants grew to a height of about 30 cm, they were subjected to heat wave stress (40 ± 1°C) for 5 days; the daily treatment time was from 9:00 to 17:00 h; the temperature for the remaining time period was kept the same as the normal day temperature. After 5 days, the plants were returned to normal temperature and the other conditions remained unchanged. For the control group (Group CK), the day and night temperatures, photoperiod, and light intensity were the same as those of the experimental group, the CO_2_ concentration was 370 ppm. The plants grew under such conditions throughout the study. Thereafter, the adults of *O. communa*, aged 5–8 days, were attached to the plants to lay eggs, and the hatched larvae were directly raised on the same plant to the pupal and adult stages. One day after eclosion, the male adults were chosen to feed alone (still in the corresponding incubator) until they were sexually mature (2–4 days after eclosion). The unmated male adults were taken for anatomical examination. In particular, because it is not possible to tell the sex of *O. communa* at the larval or pupal stages, the males had to be identified in the adult stage.

### Sample Preparation and RNA Extraction

The reproductive tract of *O. communa* males is mainly dominated by a pair of testes and male accessory glands (Supplementary Figure S1). In the present study, the testes (TE) and the male accessory glands (MAG) were sampled separately. For each tissue type, three independent replicates representing tissues pooled from 30 individuals were collected. The tissues of the male adults were dissected and washed in sterile ice-cold phosphate-buffered saline (PBS) treated with 0.1% diethylpyrocarbonate (DEPC), and were then immediately frozen and stored at −80°C for subsequent RNA extraction.

Total RNA was extracted with Trizol reagent (Life Technologies, United States) according to the manufacturer’s instructions. RNA quality was examined as follows: RNA degradation and contamination was monitored on 1% agarose gels; RNA purity was checked using the NanoPhotometer^®^ spectrophotometer (IMPLEN, CA, United States); RNA concentration was measured using Qubit^®^ RNA Assay Kit in Qubit^®^ 2.0 Fluorometer (Life Technologies, CA, United States); RNA integrity was assessed using the RNA Nano 6000 Assay Kit with the Agilent Bioanalyzer 2100 system (Agilent Technologies, CA, United States).

### cDNA Library Construction and RNA-Seq Analysis

The RNA of the required quality was sent to Beijing Genomics Institute in Wuhan for cDNA library construction and RNA- sequencing (RNA-seq). A total of 3 μg RNA per sample was used as the input material for RNA sample preparation. Sequencing libraries were generated using NEBNext^®^ Ultra^TM^ RNA Library Prep Kit for Illumina^®^ (NEB, United States) following manufacturer’s recommendations and index codes were added for attributing sequences to each sample. Briefly, mRNA was purified from total RNA using poly-T oligo-attached magnetic beads. Fragmentation was carried out using divalent cations under elevated temperature in NEBNext First Strand Synthesis Reaction Buffer (5X). First strand cDNA was synthesized using random hexamer primer and M-MuLV Reverse Transcriptase (RNase H^–^) (NEB, United States). The synthesis of second strand cDNA was subsequently performed using DNA polymerase I, and it was treated with RNase H. The remaining overhangs were converted into blunt ends via exonuclease/polymerase activities. After adenylation of the 3′-ends of the DNA fragments, NEBNext Adaptors with hairpin loop structure were ligated to prepare for hybridization. To select cDNA fragments, preferentially those that were 300 bp in length, the library fragments were purified with AMPure XP system (Beckman Coulter, Beverly, MA, United States). Thereafter, 3 μL USER Enzyme (NEB, United States) was mixed with size-selected, adaptor-ligated cDNA at 37°C for 15 min, followed by incubation for 5 min at 95°C before PCR. Subsequently, PCR was performed with Phusion High-Fidelity DNA polymerase, Universal PCR primers, and Index (X) primer. The amplified PCR products were purified (AMPure XP system) and the library quality was assessed on the Agilent Bioanalyzer 2100 system.

The clustering of the index-coded samples was performed on a cBot Cluster Generation System using TruSeq PE Cluster Kit v3-cBot-HS (Illumina) according to the manufacturer’s instructions. After cluster generation, the library preparations were sequenced on an Illumina Hiseq platform (Hiseq X Ten, Illumina, Inc., San Diego, CA, United States) and paired-end reads (150 bp) were generated.

### Data Processing, Assembly, and Annotation

Raw data (raw reads) in the fastq format were first processed through in-house perl scripts. In this step, clean data (clean reads) were obtained by removing the reads containing the adapter, reads containing ploy-N, and low quality reads from raw data. At the same time, Q20, Q30, GC-content, and sequence duplication levels of the clean data were calculated. All the downstream analyses were based on clean data of high quality. To obtain an integrated transcript set, all the clean data were put together and subjected to a combined assembly strategy. The transcript sequence obtained by splicing with Trinity was used as a reference sequence for subsequent analysis. The longest transcript of each gene was selected as Unigene for subsequent analysis. The left files (read 1 files) from all samples were pooled into one big left.fq file, and right files (read 2 files) into one big right.fq file. Transcriptome assembly was accomplished based on the left.fq and right.fq using Trinity (Grabherr et al., 2011) with min_kmer_cov set to 2 by default and all other parameters set default.

Seven databases were used to annotate the gene function; these were: Nr (NCBI non-redundant protein sequences, NCBI blast 2.2.28+, *E*-value = 1E-5^1^); Nt (NCBI nucleotide sequences, NCBI blast 2.2.28+, *E*-value = 1E-5^2^); Pfam (Protein family^3^, HMMER 3.0 package, hmmscan, *E*-value = 0.01); KOG/COG (Clusters of Orthologous Groups of proteins^4^, NCBI blast 2.2.28+, *E*-value = 1E-3); Swiss-Prot (a manually annotated and reviewed protein sequence database^5^, NCBI blast 2.2.28+, *E*-value = 1E-5); KO (KEGG Ortholog database^6^, KAAS (version r140224), KEGG Automatic Annotation Server, *E*-value = 1E-10] (Kanehisa et al., 2008); and GO (Gene Ontology^7^, Blast2GO v2.5, *E*-value = 1E-6) (Gotz et al., 2008).

Long non-coding RNAs (lncRNA) were predicted using CPC (Coding Potential Calculator^8^). In order to reduce the false positive rate in predicting lncRNA, the final results would be given on removing the transcript annotated by Swiss-Prot and Pfam database.

### SNP and InDel Calling

Picard-tools v1.41^9^ and samtools v0.1.18 (Li, 2011) were used to sort and remove duplicated reads, and merge the bam alignment results for each sample. GATK2 software (Mckenna et al., 2010) was used to perform SNP and InDel calling. The raw vcf files were filtered with the GATK standard filter method and other parameters [clusterWindowSize: 10; MQ0 ≥ 4 and (MQ0/(1.0^∗^DP)) > 0.1; QUAL < 10; QUAL < 30.0, QD < 5.0, HRun > 5], and only SNPs with a distance >5 were retained.

### Analysis of Gene Expression Patterns

Gene expression levels were estimated by RSME (Li and Dewey, 2011) for each sample. RSEM counts bowtie’s results, clean data were mapped back onto the assembled transcriptome, and readcount for each gene was obtained from the mapping results. Read counts were converted to FPKM (expected number of Fragments Per Kilobase of transcript sequence per Millions base pair sequenced) values, and expression values were calculated in terms of FPKM (FPKM > 0.3) (Trapnell et al., 2010). For the same samples with biological replicates, differential expression analysis for the two conditions was performed using the DESeq R package^10^ (Anders and Huber, 2010). DESeq provides statistical routines for determining differential expression in the digital gene expression data using a model based on the negative binomial distribution. The resulting *P*-values were adjusted using the Benjamini and Hochberg’s approach for controlling the false discovery rate. Genes with an adjusted *P*-value < 0.05 estimated by DESeq were assigned as differentially expressed. Significance tests for each gene were conducted for “TE vs. TEck,” “MAG vs. MAGck.”

### Gene Ontology Enrichment and KEGG Pathway Analyses

Gene Ontology (GO) enrichment analysis of the differentially expressed genes (DEGs) was implemented by the GOseq R packages based on Wallenius non-central hyper-geometric distribution (Young et al., 2010), which can adjust for gene length bias in DEGs. KEGG (Kanehisa et al., 2008) is a database resource for understanding high-level functions and utilities of the biological system, such as the cell, the organism, and the ecosystem, from molecular-level information, especially large-scale molecular datasets generated by genome sequencing and other high-throughput experimental technologies^6^. KOBAS (Mao et al., 2005) software was used to test the statistical enrichment of the DEGs in KEGG pathways.

### Quantitative Real-Time PCR (qRT-PCR) Validation

To validate the results of differential expression obtained in the RNAseq analysis, 10 DEGs were analyzed by quantitative real-time PCR (qRT-PCR), with three biological replicates, including 4 DEGs in “MAG vs. MAGck” and 6 DEGs in “TE vs. TEck.” Total RNA was extracted from the samples mentioned above. Thereafter, 1,000 ng of total RNA was reverse transcribed in a 20 μL reaction containing 5× TransScript All-in-One SuperMix for Real time (TransGen Biotech, Beijing). All the specific primers were 18–20 bp and amplified 80–150 bp PCR products; the Tm for the primes ranged from 58 to 60°C. The primers are listed in Supplementary Table S1. A reference housekeeping gene (ribosomal protein L4, not yet published) was used for normalization. The reaction mixture (final volume of 20 μL) consisted of 1 μL cDNA, 10 μL SYBR Green Master Mix, 0.4 μL of each specific primer (10 μM), and 8.2 μL ddH_2_O. The thermal-cycler program was as follows: 5 min at 95°C for the initial denaturation, followed by 40 cycles of 10 s at 95°C and 34 s at 60°C. The 2^–ΔΔ*CT*^ method was used to estimate the changes in the expression between the two treatments (Livak and Schmittgen, 2001). The data were analyzed by the student *t*-test with SPSS 22.0 software (SPSS Inc., Chicago, IL, United States). The results were expressed as means ± SE.

## Results

### Establishment of the Male *O. communa* Transcriptome From the TE and MAG

The transcriptomes of TE and MAG from different treatment groups were sequenced in triplicates. After preprocessing the reads, a total of 72.3 GB of clean data was acquired. The Q20 and Q30 for all the samples were ≥96 and >91%, respectively. The GC content was about 40% (Supplementary Table S2). Because of the absence of a reference genome, the obtained clean reads were assembled to get a reference sequence for subsequent analysis. Overall, 67,205 unigenes were generated from the transcriptome, which included 21,928 coding and 45,277 long non-coding genes. The mean length of the unigenes was 768 bp, with N50 and N90 lengths of 1,468 bp and 274 bp, respectively (Table 1). Sequence length distribution analysis showed that 52.01% of the unigenes were between 301 and 2,000 bp in size, and unigenes less than 301 bp and greater than 2,000 bp accounted for 39.09 and 8.9% of the total number, respectively (Supplementary Figure S2).

**TABLE 1:** Statistics of the final assembly and prediction of coding genes.

Total length (bp)	Mean length (bp)	N50	N90	Total number (≥200 bp)	Total number (≥1 kb)	Coding genes	Long non-coding genes
51633129	768	1468	274	67205	13674	21928	45277

### Gene Function Annotation

The transcriptome gene function was annotated using seven databases (Supplementary Table S3). A total of 28,369 unigenes (42.21% of the total unigenes) were annotated in at least one of the databases. Maximum number of genes (34.4%) were annotated in the NR database (Supplementary Table S3): most of the sequences (41.2%) matched with those of the model organism, *Tribolium castaneum*, followed by those of *Dendroctonus ponderosae* (12.8%), *Lasius niger* (4.2%), *Acyrthosiphon pisum* (3.8%), *Vollenhovia emeryi* (2.5%); the remaining 35.5% of unigenes gave BLAST hits with sequences from other species (Supplementary Figure S3). Besides, several unigenes were annotated using the sequences in the NT, KO, SwissProt, and PFAM databases (Supplementary Table S3).

After the GO annotation of genes, they were grouped into three main categories, namely “Biological process,” “Cellular process,” and “Molecular function” (Supplementary Figure S4). Among the processes, based on the KOG database, 10.72% of the unigenes were categorized into 26 different functional groups (Supplementary Figure S5). After KO annotation of the genes, they were classified according to the KEGG metabolic pathways they participated in. These genes belonged to five branches (“Cellular Processes,” Environmental Information Processing,” “Genetic Information Processing,” “Metabolism,” and “Organismal Systems”) (Supplementary Figure S6).

### Putative SNPs and InDels

A total of 203411 SNPs and 31254 InDels were identified in this study. The distribution of SNPs in each sample is shown in Supplementary Table S4. The number and percentage of SNPs in each coding and non-coding region of each sample were calculated. Additionally, the number and percentage of synonymous and non-synonymous mutations per sample were identified. These molecular markers, SNPs and InDels, might be useful for further studies to detect the migration and diffusion of *O. communa*. The identity of the predicted molecular markers should be validated in future research to ensure their accuracy.

### Gene Expression Analysis Across Different Treatments

**FIGURE 1:**
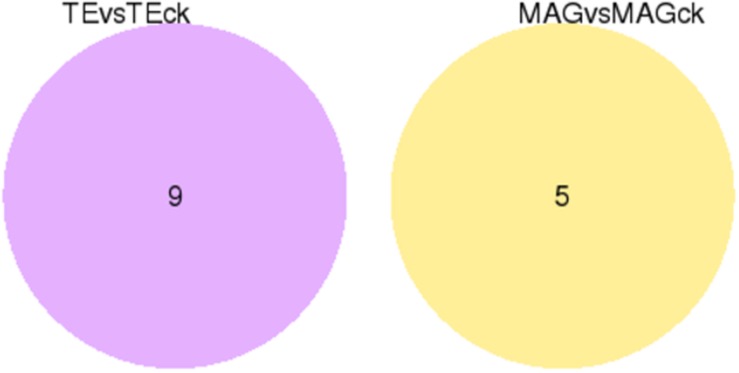
Venn diagram for the differentially expressed genes identified in different comparisons.

**FIGURE 2:**
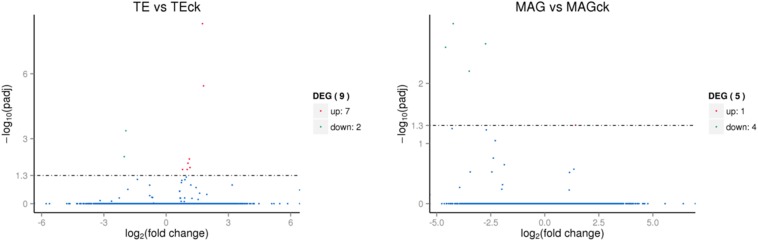
Significantly up- and down-regulated differentially-expressed genes (DEGs) between the tissues. The abscissa represents the fold-change in the expression of genes in different samples; the ordinate represents the statistical significance of the change in gene expression; the smaller the corrected *p*-value, the larger is the -log10 (corrected *p*-value), that is, more remarkable is the difference. The scatter points in the figure represent individual genes, the blue dots indicate genes with no significant differences, the red dots indicate up-regulated genes with significant differences, and the green dots indicate down-regulated genes with significant differences.

For identifying DEGs among the tissues in the different treatment groups, the FPKM conversion for each read count was performed, and the unigenes with FPKM values above 0.3 were selected. The results showed that 10,296 unigenes were expressed in all the samples, and there were several unigenes specifically expressed in each sample (on account of 3 replicates) of TE, TEck, MAG, and MAGck groups (Supplementary Table S5). The overall view of gene expression in each sample is presented in the FPKM boxplot (Supplementary Figure S7). The discreteness of gene expression indicates the degree of difference between samples. Venn diagrams were used to present the number of common and unique differential genes between different combinations of comparisons (Figure 1). The relationship between qvalue and log2 (foldchange) of the unigenes and the significantly up- and down-regulated DEGs between two tissues were revealed using the volcano plot (Figure 2). Unexpectedly, only nine and five DEGs were identified in the comparisons of TE with TEck and of MAG with MAGck, respectively (Figure 1). Among the nine DEGs, seven were up-regulated and two were down-regulated; among the five DEGs, one was up-regulated and four were down-regulated (Figure 2).

This study focused on the indirect effects of the elevated atmospheric CO_2_ levels associated with heat waves on the male reproductive tract of *O. communa*. The transcriptional changes in the TE vs. TEck and MAG vs. MAGck comparisons could reflect these effects. Among the DEGs, one odorant binding protein gene related to host plants that was up-regulated. Three DEGs related to the adjustment of energy metabolism were annotated, among which the unigene encoding putative sugar transporter 2 in the MAG vs. MAGck comparison was down-regulated and the other two DEGs encoding putative glycosyl hydrolase and alpha-esterase in the TE vs. TEck comparison were up-regulated. There were three stress-related DEGs including two kinds of predicted facilitated trehalose transporters, Tret1 (down-regulated) and an extracellular Cu/Zn-superoxide dismutase (up-regulated). A putative juvenile hormone, epoxide hydrolase 2 (up-regulated) related to reproduction was also identified. Six DEGs were not annotated using the NR database; however, the unigene, Oc27982_g1, was identified as defensin-related using PFAM; the unigene, Oc40935_g1, was annotated as acyltransferase using PFAM, which plays important roles in gene expression, metabolism, and signaling; the unigene, Oc45230_g1, was also annotated as an odorant binding protein gene using PFAM; the unigene, Oc41705_g2, was annotated as multidrug resistance-associated protein using SwissProt, PFAM, and KOG, indicating that this gene could be detoxification-related; the unigene, Oc37599_g1, was annotated as a transporter in other databases, which may be detoxification-related; the unigene, Oc37929_g1, was annotated as aldose reductase using SwissProt, which might play a role in the protective response to feeding on common ragweed exposed to heat wave and elevated CO_2_ concentrations (Table 2). The function of Oc27982_g1, Oc40935_g1, Oc41705_g2, Oc37599_g1, and Oc37929_g1 were all related to poisonous food-utilization.

**TABLE 2:** Differentially expressed genes identified in the TE vs. TEck and MAG vs. MAGck comparisons.

**Gene ID**	**NR Description**	**TE_Readcount**	**TEck_Readcount**	**Log2FoldChange**	**Padj**

Oc33781_g1	Minus-C odorant binding protein 4	3321.071	986.2709	1.7516	4.84E-09
Oc36319_g1	Putative glycosyl hydrolase	315.841	90.68502	1.8003	3.61E-06
Oc42539_g1	PREDICTED: facilitated trehalose transporter Tret1	51.6886	198.3142	−1.9399	0.000427
Oc27982_g1	–	18.76356	76.12178	−2.0204	0.006726
Oc28991_g1	Extracellular Cu/Zn-superoxide dismutase	283.3689	130.6129	1.1174	0.00865
Oc40935_g1	Uncharacterised protein	294.4951	142.0578	1.0518	0.013529
Oc44830_g2	Putative alpha-esterase	217.3302	97.95882	1.1496	0.021473
Oc45230_g1	Uncharacterised protein	307.2207	152.0575	1.0147	0.026302
Oc45685_g1	Putative juvenile hormone epoxide hydrolase 2	3856.158	2228.029	0.7914	0.026302

**Gene ID**	**NR description**	**MAG_Readcount**	**MAGck_Readcount**	**Log2FoldChange**	**Padj**

Oc41705_g2	Hypothetical protein	1.778576	33.55527	−4.2377	0.001018
Oc37599_g1	Unknown	8.560656	57.11945	−2.7382	0.002196
Oc38167_g1	Putative sugar transporter 2, partial	17.8918	430.7275	−4.5894	0.00251
Oc43123_g1	PREDICTED: facilitated trehalose transporter Tret1	3.007809	33.93026	−3.4958	0.006256
Oc37929_g1	Unknown	177.0935	65.9373	1.4253	0.049541

### KEGG Enrichment of DEGs

**FIGURE 3:**
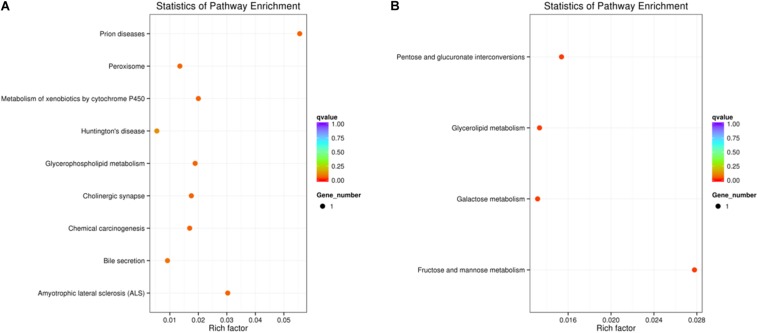
Scatter plot for KEGG pathway enrichment of differentially expressed genes. The vertical axis represents the path name and the horizontal axis represents the Rich factor corresponding to the path. The size of the *q*-value is represented by the color of the dot. The smaller the *q*-value is, the closer the color is to red. The number of differential genes contained in each route is represented by the size of the dot. **(A)** TE vs. TEck. **(B)** MAG vs. MAGck.

All the DEGs were mapped to the terms for enrichment analysis in the KEGG database. In the comparison of TE vs. TEck, 3 DEGs (Oc28991_g1, Oc45685_g1, and Oc44830_g2) were involved in 9 pathways, which were up-regulated. Oc28991_g1 was enriched in “prion diseases,” “amyotrophic lateral sclerosis (ALS),” “peroxisome” and “huntington’s disease.” Oc45685_g1 was enriched in “metabolism of xenobiotics by cytochrome P450,” “chemical carcinogenesis,” and “bile secretion.” Oc44830_g2 was enriched in “glycerophospholipid metabolism” and “cholinergic synapse.” In the comparison of MAG vs. MAGck, only Oc37929_g1 (up-regulated) was enriched in “fructose and mannose metabolism” “pentose and glucuronate interconversions” “glycerolipid metabolism,” and “galactose metabolism” pathways (Figures 3A,B).

### Validation of Transcriptome Data Using qPCR

**FIGURE 4:**
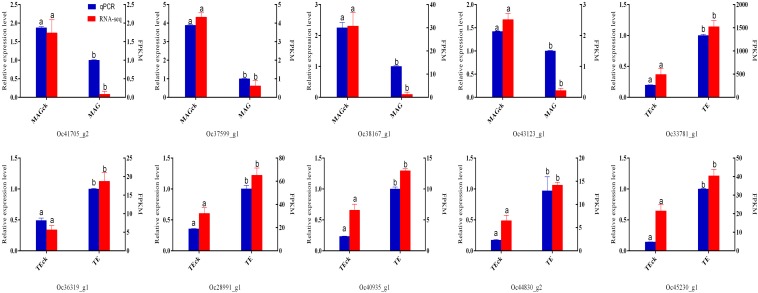
Expression levels of differentially expressed genes as analyzed by RNA-seq and qPCR.

Among these 10 DEGs, the gene expression patterns analyzed by qPCR were consistent with the results of the FPKM method (Figure 4), indicating that the transcriptome analysis results are reliable.

### Seminal Fluid Proteins of Other Insects Reported in This Study

Recently, many seminal fluid proteins were identified in insects, but most of them have no names or their functions remain unclear. Based on the annotation of the transcriptome, the unigenes were investigated, which were annotated as the usual seminal fluid proteins (Table 3). Only one unigene was annotated for “protein outspread,” “prophenoloxidase,” and “unitz-like protease inhibitor.” There were more than two unigenes annotated for other seminal fluid proteins, which need further confirmation.

**TABLE 3:** The unigenes annotated as the usual seminal fluid proteins in other insects.

Reported seminal fluid proteins	Number of unigenes
Serine protease inhibitor	(number: 35) Oc50230_g1, Oc40958_g1, Oc40175_g1, Oc32327_g1, Oc39266_g2, Oc41818_g2, Oc39619_g1, Oc40450_g1, Oc36330_g3, Oc35303_g1, Oc5184_g2, Oc43096_g5, Oc61813_g1, Oc54131_g1, Oc40980_g2, Oc44411_g2, Oc42986_g5, Oc45048_g4, Oc40924_g7, Oc46205_g1, Oc41429_g2, Oc58972_g1, Oc39090_g1, Oc43313_g1, Oc29486_g1, Oc37233_g2, Oc45867_g3, Oc45893_g1, Oc44847_g1, Oc43803_g1, Oc54731_g1, Oc27882_g1, Oc43943_g3, Oc30765_g1, Oc43748_g2
Inosine triphosphate pyrophosphatase	Oc54086_g1, Oc36912_g1
prophenoloxidase	Oc41463_g1, Oc40687_g1
Sex peptide	Oc39226_g1, Oc25143_g1, Oc14379_g1, Oc47450_g1
Accessory gland-specific peptide	Oc38938_g4, Oc37240_g1, Oc45993_g2
Acyl carrier protein	Oc38619_g1, Oc20011_g2, Oc59854_g1
Accessory gland secretory protein	Oc36414_g2, Oc33429_g1
Trypsin inhibitor	(number: 17) Oc40339_g2, Oc7921_g1, Oc45020_g2, Oc44386_g1, Oc41286_g1, Oc44917_g1, Oc42064_g1, Oc38028_g1, Oc21841_g1, Oc44895_g1, Oc45174_g1, Oc12782_g2, Oc9428_g1, Oc44955_g1, Oc12782_g1, Oc30607_g1, Oc41546_g5
Protein outspread	Oc38683_g1
Heat shock protein	(number: 35) Oc40056_g1, Oc48899_g1, Oc45298_g1, Oc42711_g6, Oc3637_g1, Oc36919_g1, Oc34610_g1, Oc46046_g4, Oc60165_g1, Oc39925_g1, Oc36955_g2, Oc36955_g1, Oc45569_g1, Oc44651_g4, Oc57392_g1, Oc52330_g1, Oc36345_g1, Oc48015_g1, Oc63820_g1, Oc38984_g1, Oc23373_g1, Oc25724_g1, Oc41619_g1, Oc40275_g4, Oc39129_g1, Oc54499_g1, Oc40902_g1, Oc22529_g1, Oc39150_g1, Oc50596_g1, Oc18118_g1, Oc42720_g2, Oc45906_g1, Oc41058_g1, Oc4267_g1, Oc40569_g1, Oc62768_g1, Oc37460_g1, Oc54241_g1, Oc37879_g2, Oc63216_g1, Oc53882_g1, Oc45930_g1, Oc42860_g9, Oc34266_g1, Oc41060_g1, Oc32701_g2, Oc45881_g2, Oc39370_g3, Oc41544_g1, Oc42148_g1, Oc37476_g1, Oc54348_g1, Oc32701_g1, Oc31794_g1, Oc36618_g1, Oc58935_g1
Angiotensin-converting enzyme	(number: 7) Oc45845_g1, Oc38060_g1, Oc37499_g1, Oc43314_g1, Oc42324_g1, Oc39440_g1, Oc45009_g2
Prophenoloxidase	Oc40687_g1
Unitz-like protease inhibitor	Oc23657_g1
Juvenile hormone binding	Oc38374_g1, Oc38374_g2
Regucalcin	(number: 7) Oc36186_g1, Oc8883_g1, Oc37602_g1, Oc42927_g1, Oc47382_g1, Oc40779_g2, Oc45476_g2, Oc44619_g1, Oc24349_g1
……	……

## Discussion

The use of high-throughput transcriptome technologies enables to investigate the genetic responses that organisms deploy in alternative environments (Roy et al., 2010). So far, RNA-seq was employed to evaluate the changes induced by different treatments (Zheng et al., 2013; Alfonso-Parra et al., 2016; Wu et al., 2019). In the present study, the RNA-seq data was analyzed to explain the effect of climate change on *O. communa*. The expression profiles between TE and TEck, and between MAG and MAGck represent the differences in the reproductive tract of males upon feeding common ragweed exposed to different conditions, which simulate the closest rearing conditions prevalent in nature. Based on the statistical analysis, differently expressed genes were identified.

Two DEGs were annotated as putative sugar transporter 2 (Oc38167_g1) and putative glycosyl hydrolase (Oc36319_g1). Oc44830_g2 was enriched in “glycerophospholipid metabolism” and “cholinergic synapse” pathways. The proteins participate in the decomposition and transport of macromolecular substances. This suggests that differentially expressed energy metabolism-related genes may be involved in regulation of reproduction. Odorant binding proteins are well-known to facilitate the transfer of apolar odorants in the aqueous antennal perilymph during pheromone reception (Vogt et al., 1991; Shen et al., 2019). Among the DEGs identified for the TE vs. TEck comparison, two odorant binding proteins (Oc33781_g1 and Oc45230_g1) were identified. They may be related to the response of *O. communa* to the host plant exposed to high CO_2_ concentration and heat waves. Similarly, two odorant binding proteins were reported to affect the taste perception and host-plant preference in *Drosophila sechellia* (Matsuo et al., 2007). However, little is known about the regulatory mechanism for the insects’ post mating behavior. A DEG, Oc45685_g1, was identified as putative juvenile hormone epoxide hydrolase 2. Juvenile hormone (JH) is an essential hormone for insects. It is called “insect growth regulator” (Staal, 1975) and has central roles in the regulation of insect development and reproduction (Laufer et al., 1987). The juvenile hormone, epoxide hydrolase, regulates insect JH titre along with JH esterase (Touhara and Prestwich, 1993). Oc45685_g1 was up-regulated in TE with respect to its level in TEck, indicating that the treatment to males may affect their development and reproduction. Indeed, in a study on *T. castaneum*, researchers found that the MAG size and SFP amount in JH-deficient males decreased and they acted with less vigor in the mating behavior, there was poor sperm transfer, and less production of eggs and offspring (Parthasarathy et al., 2009).

Trehalose plays a much more complex role in insect physiology than just being an energy store (Jungreis, 1980). The trehalose concentration can regulate taste receptors and the central nervous system, which affects the insects’ food selection and feeding behavior (Thompson Nelson, 2003). It can also act as a stabilizer of proteins to protect insects from harsh environments (Wyatt, 1967). The two DEGs, trehalose transporter Tret1 (Oc42539_g1 and Oc43123_g1), which were down-regulated, might be responsible for the fact that the population showed an obvious declining trend in the hottest days. Superoxide dismutase (SOD) is a catalyser in the disproportionation which reduces oxygen to generate oxygen and hydrogen peroxide. SOD has been widely discovered in the cells of various organisms. It can specifically scavenge superoxide anion and protect the body from oxidative damage (Xu, 2013). Cu/Zn-superoxide dismutase is one enzyme of SOD. Mittapalli et al. (2007) suggested that the induced expression of superoxide dismutase gene, in the midgut of *Mayetiola destructor*, caused a protective response to oxidative damage from the food. In this study, Oc28991_g1, encoding Cu/Zn-superoxide dismutase, may also play an important role in protective response to oxidative damage while feeding the beetles on the weed. Different expressions of these three DEGs help to mitigate the indirect stresses created by high CO_2_ concentration combined with the heat waves.

For *O. communa*, successful mating is necessary for producing the offspring. Numerous SFPs are major effectors for a range of female postmating responses related to fertility. They are transferred to females with sperm during mating (Avila et al., 2011). Therefore, identification of SFPs is the primary step to understand the changes in reproduction. Many researchers predicted the insect SFPs using comparative transcriptomics. McGraw et al. (2008) examined gene expression profiles of whole female *Drosophila melanogaster* at four time points following copulation to explore the function of SFPs. Gotoh et al. (2017) identified enriched genes in the sperm-storage organ relative to those in the body of queen *Crematogaster osakensis* to identify the putative SFPs. SFPs are the products of male reproductive tract secretory tissues, not only including accessory glands (Avila et al., 2011); however, in most of the studies, the accessory gland has been considered as the only organ that secretes SFPs, and these have been defined as accessory gland proteins (Acps) (Chapman and Davies, 2004; South et al., 2011; Yu et al., 2016). For rigorous experiment, the male accessory glands and testes were both sampled. If all the DEGs in this study were transferred to females as SFPs, they may be involved in changing female likehood of remating, increasing ovulation and egg-laying rates, changing female flight and feeding behavior, inducing antimicrobial activities, and modulating sperm storage parameters. An increasing number of SFPs have been identified in insects and the function of these identified SFPs include roles in processes, such as odorant binding, and stress response, in addition to some functions that remain unknown. For example, Sirot et al. (2008) and Xu et al. (2013) found odorant binding proteins were SFPs of *Aedes aegypti*. In the present study, two DEGs encoding odorant binding proteins were also revealed. Certainly, the proteins from the male reproductive tract may alter the sperm production and quality except for being effectors as SFPs. The formation of sperm involves a series of molecular and spectacular morphological changes in insect testes (Vedelek et al., 2018). Thus, reduction of sperm motility and viability may also contribute the effect of the reproduction of females.

Although SFPs evolve rapidly, the protein classes represented in seminal fluid are constrained (Mueller et al., 2005). We investigated all the unigenes in our data, using several common SFPs as keywords, and found that they all matched one or more unigenes (Table 3). We could not tell if any of the unigenes was an SFP gene, but the genes encoding SFPs of *O. communa* were certainly included in this transcriptome. In future research, the function of some representative SFPs could directly be carried out based on the existing data resources. The present study provides a new perspective on revealing the functions of SFPs. In addition, Avila et al. (2011) raised an important point that Acp (SFP) genes evolve *de novo*, perhaps from long non-coding RNA. In this study, 45277 long non-coding genes were predicted, which provide novel and broad avenues for further research.

Generally, many DEGs can be identified by comparing the transcriptomes (Wissing et al., 2014; Lin et al., 2018). However, 14 DEGs were identified in the TE vs. TEck and MAG vs. MAGck comparisons. The reasons for the decline in population could be multifaceted. The females’ post-mating response caused by SFPs and sperm motility and numbers is only one aspect, which mainly affects the number of offspring. The impact on females themselves has not been investigated. Female specific proteins, containing vitellin (VN) and vitellogenin (VG), are main nutrition resource in early stage of life for oviparous insects (Swevers et al., 2005; Zhao et al., 2018). VG proteins become the major VN proteins (Hagedorn and Kunkel, 1979). Therefore, the VG gene expression level in females is key role in determining reproductive potential. A follow-up research would be needed to confirm it. Besides, feeding on common ragweed exposed to high CO_2_ concentration and heat wave may affect other organs of *O. communa*, such as olfactory organ and midgut, which play a direct role in the development and survival of the first generation. Insects depend on olfaction to estimate the overall situation of habitats, involving individual aspects, such as the spotting of food, host, mate or prey, and group communication aspects, such as aggregation and avoidance (Fan et al., 2011). The midgut of insects is responsible for digestion and nutrient uptake and, in particular, for detoxification and oxidative stress response (Hakim et al., 2010). Therefore, under the same experimental conditions, DEGs in other tissues may also contribute to the changes in the population. The analysis of DEGs between the testes and accessory glands revealed the indirect response of the reproductive tract to high CO_2_ and heat waves at another level.

## Conclusion

In this study, we assembled the transcriptome of the testes and accessory glands from male *O. communa*, and investigated transcriptional changes under the premise of feeding different common ragweed, that were separately grown under high CO_2_ concentration and heat waves or under ambient CO_2_ concentration. The results indicated that the reproductive tract of males presented an indirect response to high CO_2_ concentration and heat waves. The DEGs related to decomposition and transport of macromolecular substances, host location, and stress response may trigger a series of post-copulatory events that detrimentally affect the development and growth of the population. Subsequently, along with future studies on females and other organs of *O. communa*, the molecular mechanism of the indirect response to high CO_2_ concentration and heat waves can be perfectly elucidated. And along with proteomic studies on mated females, SFPs of *O. communa* can be specifically identified.

## Data Availability Statement

The raw RNA sequencing data were deposited in the NCBI Sequence Read Archive (SRA) with the accession number PRJNA580170 (Temporary Submission ID: SUB6480492).

## Ethics Statement

The animal study was reviewed and approved by Ethics of Animal Experiment of Institute of Plant Protection, Chinese Academy of Agricultural Sciences.

## Author Contributions

ZZ and YL designed the experiments. XG, ZhenyT, ZhenqT, SC, and GC performed the experiments. XG, YZ, and CM analyzed the data. XG and ZZ wrote the manuscript.

## Conflict of Interest

The authors declare that the research was conducted in the absence of any commercial or financial relationships that could be construed as a potential conflict of interest.

## Footnotes

1 https://blast.ncbi.nlm.nih.gov/Blast.cgi?PROGRAM=blastp&PAGE_TYPE=BlastSearch&LINK_LOC=blasthome

2 https://blast.ncbi.nlm.nih.gov/Blast.cgi?PROGRAM=blastn&PAGE_TYPE=BlastSearch&LINK_LOC=blasthome

3 http://pfam.xfam.org/

4 http://www.ncbi.nlm.nih.gov/COG/

5 http://www.ebi.ac.uk/uniprot/

6 http://www.genome.jp/kegg/

7 http://www.geneontology.org/

8 http://cpc2.cbi.pku.edu.cn

9 http://broadinstitute.github.io/picard

10 https://www.huber.embl.de/users/anders/DESeq/

## Supplementary Material

FIGURE S1 Testes and accessory glands from male Ophraella communa.

FIGURE S2 Sequence-length distribution of unigenes assembled from transcripts.

FIGURE S3 Species distribution map on alignment with non-redundant (NR) database.

FIGURE S4 Gene ontology annotation of the unigenes.

FIGURE S5 KOG classification chart for the unigenes.

FIGURE S6 Classification of KEGG metabolic pathways. **(A)** Cellular processes; **(B)** environmental information processing; **(C)** genetic information processing; **(D)** metabolism; **(E)** organismal systems.

FIGURE S7 FPKM distribution of the tissues. The abscissa shows the sample name, the ordinate is log10(FPKM+1). MAG and TE: the accessory gland and testis from *Ophraella communa* males fed on ragweed exposed to elevated CO_2_ concentrations and heat wave. MAGck and TEck: the accessory gland and the testis from *O. communa* males fed on ragweed exposed to the ambient CO_2_ concentration.

TABLE S1 Primers used in qRT-PCR.

TABLE S2 Summary of the output quality of sequencing data.

TABLE S3 Statistics of the final assembly and prediction of coding genes.

TABLE S4 Distribution of single nucleotide polymorphisms (SNPs) in each sample.

TABLE S5 Statistical table of FPKM values for all the samples.
