# Supplementary figures and images for: Transcriptome Analysis of Ophraella communa Male Reproductive Tract in Indirect Response to Elevated CO2 and Heat Wave
Source: Front Physiol. 2020 May 5;11:417. doi: 10.3389/fphys.2020.00417 (PMC7215069; doi:10.3389/fphys.2020.00417)

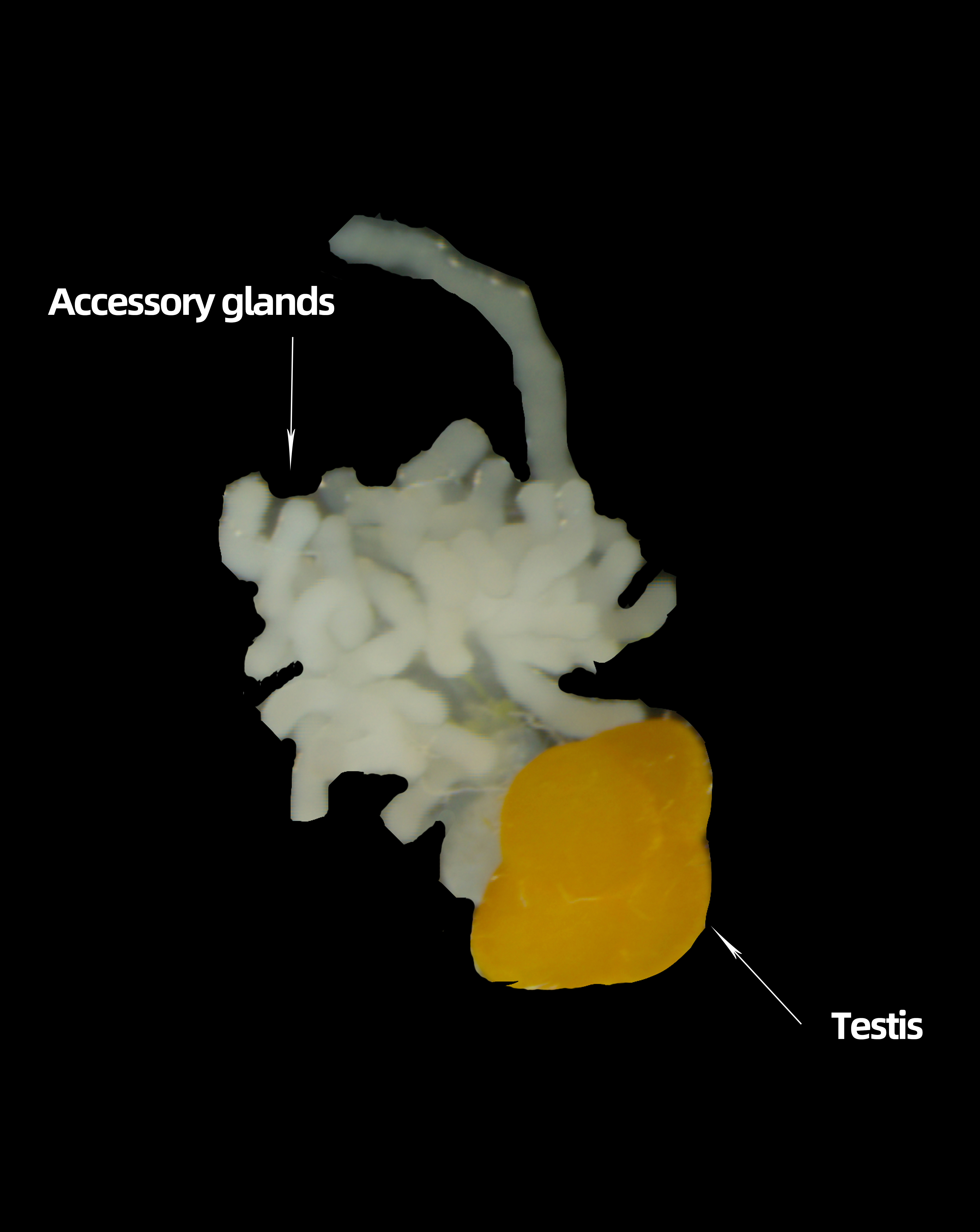

Supplement: FIGURE S1 — Testes and accessory glands from male Ophraella communa. [file Image_1.JPEG]

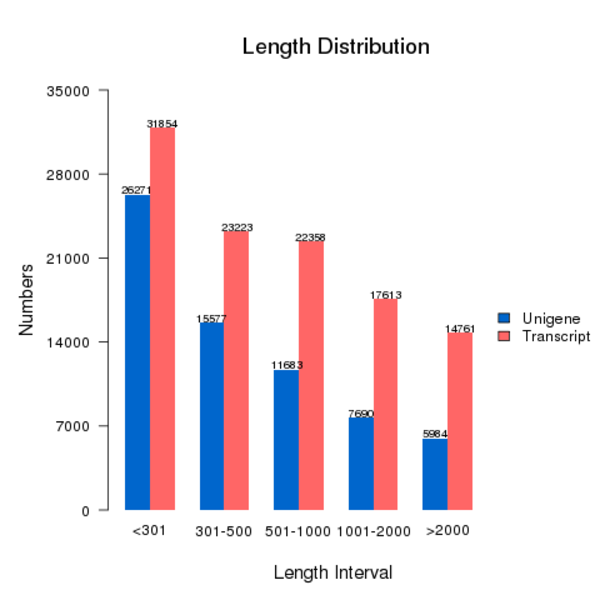

Supplement: FIGURE S2 — Sequence-length distribution of unigenes assembled from transcripts. [file Image_2.PNG]

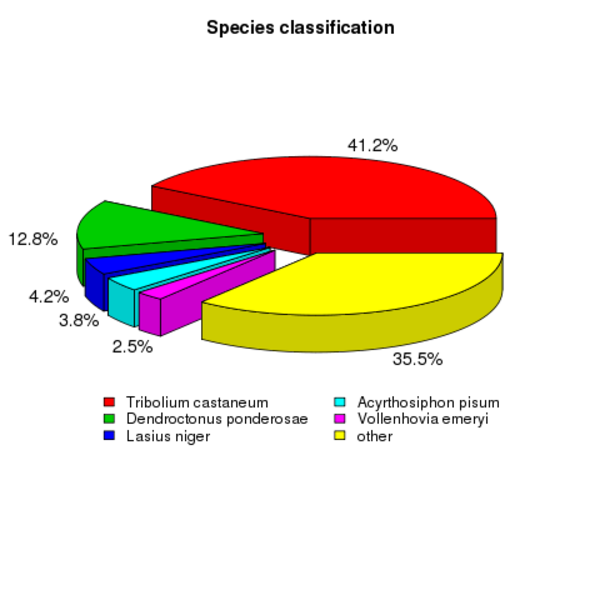

Supplement: FIGURE S3 — Species distribution map on alignment with non-redundant (NR) database. [file Image_3.PNG]

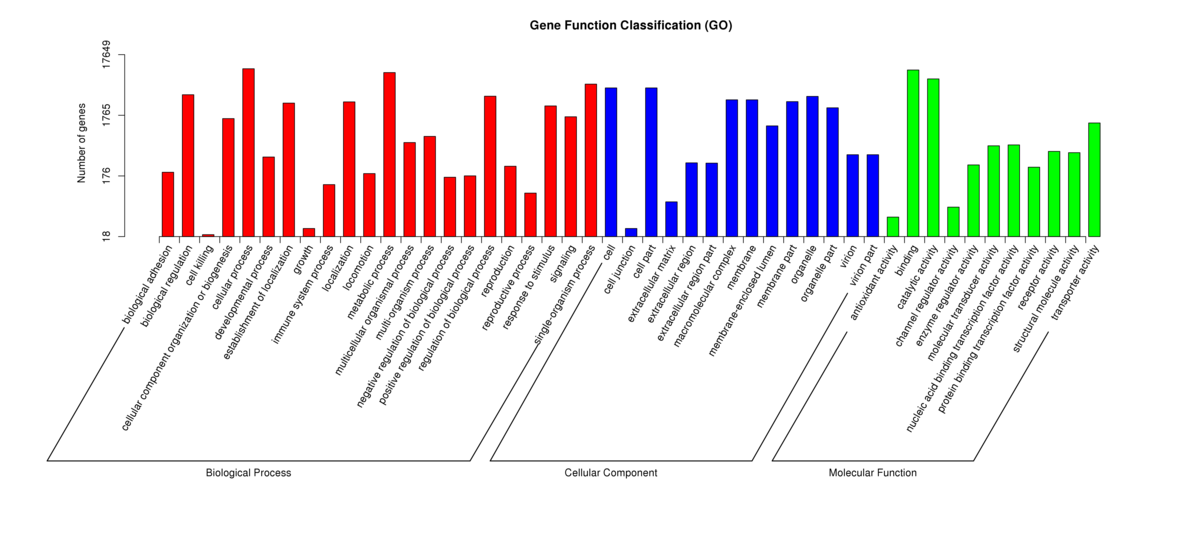

Supplement: FIGURE S4 — Gene ontology annotation of the unigenes. [file Image_4.PNG]

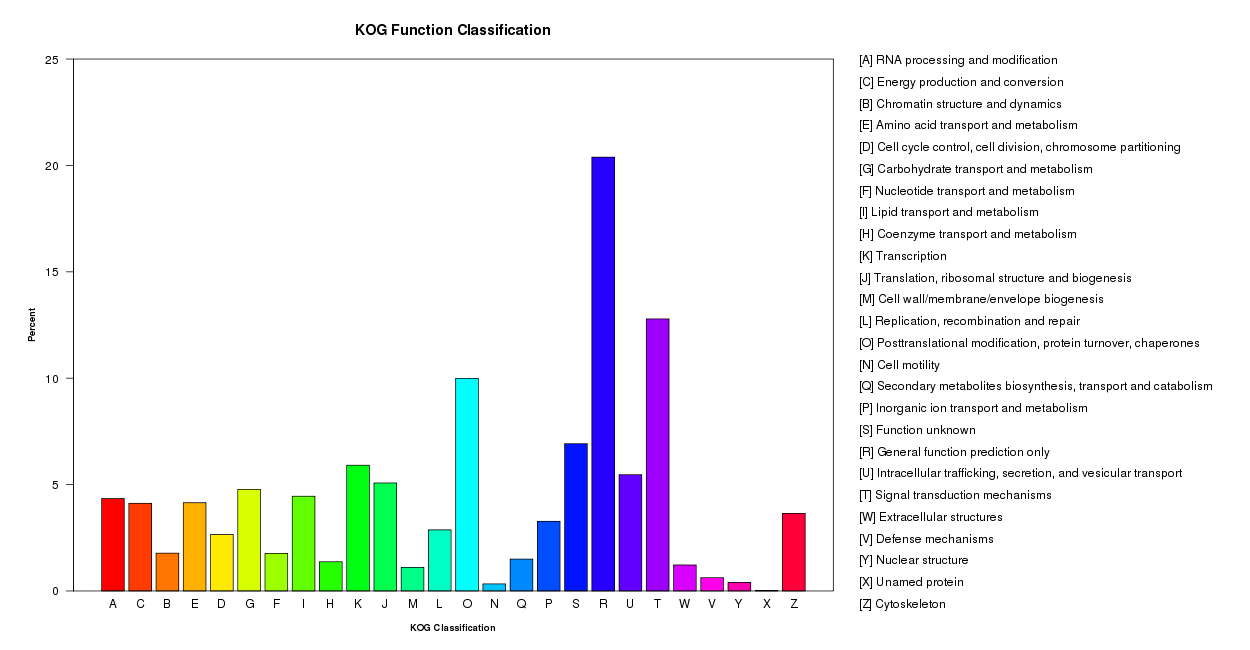

Supplement: FIGURE S5 — KOG classification chart for the unigenes. [file Image_5.PNG]

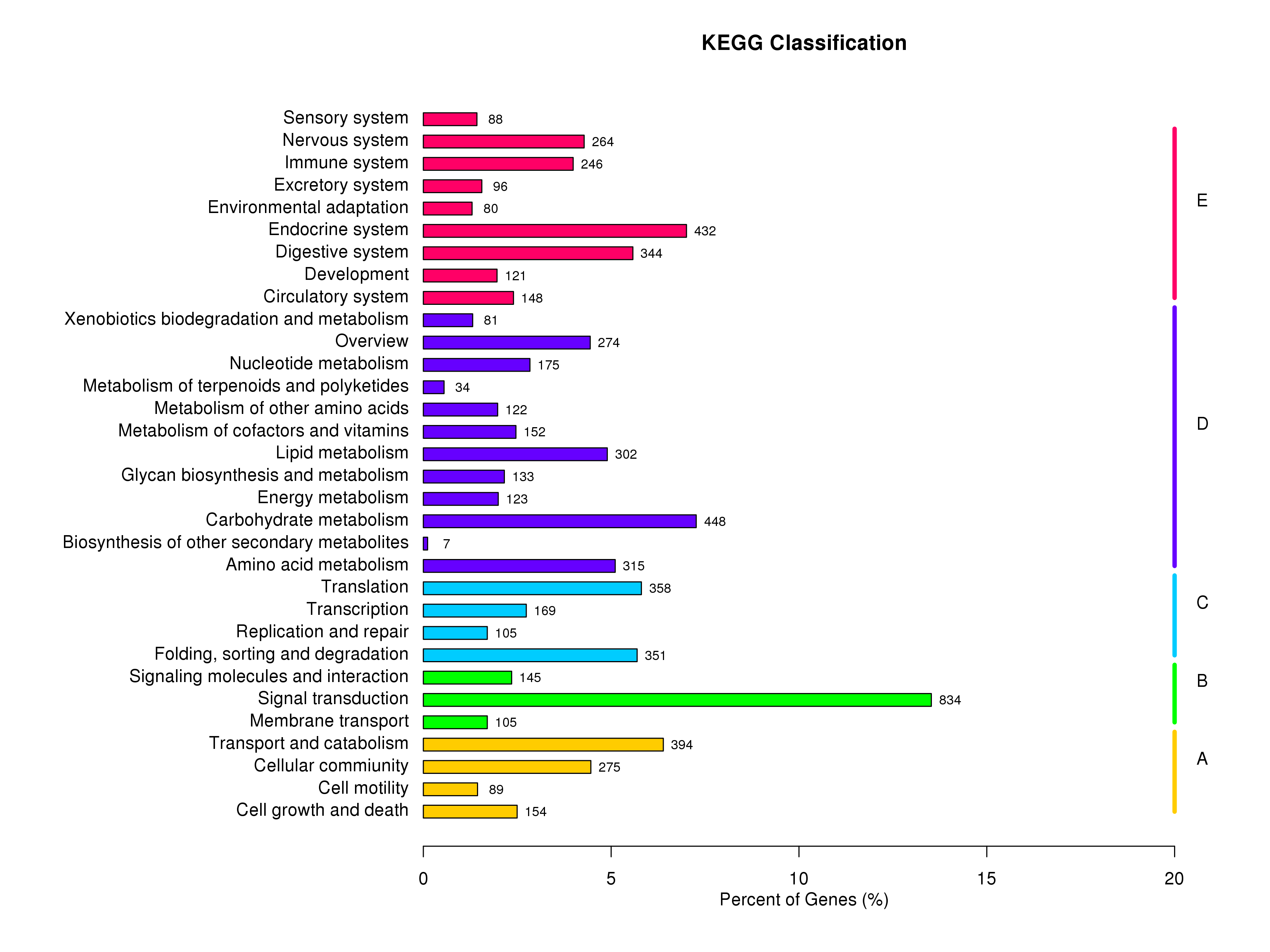

Supplement: FIGURE S6 — Classification of KEGG metabolic pathways. (A) Cellular processes; (B) environmental information processing; (C) genetic information processing; (D) metabolism; (E) organismal systems. [file Image_6.PNG]

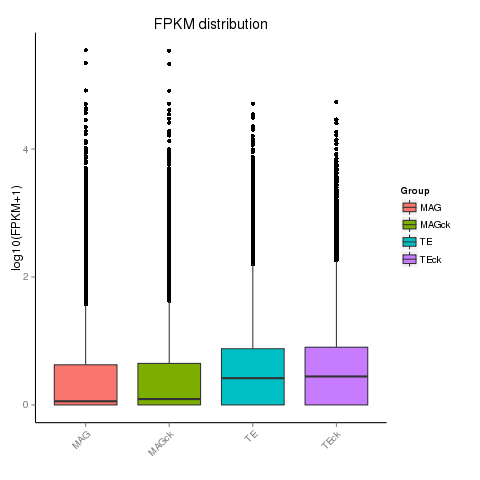

Supplement: FIGURE S7 — FPKM distribution of the tissues. The abscissa shows the sample name, the ordinate is log10(FPKM+1). MAG and TE: the accessory gland and testis from Ophraella communa males fed on ragweed exposed to elevated CO2 concentrations and heat wave. MAGck and TEck: the accessory gland and the testis from O. communa males fed on ragweed exposed to the ambient CO2 concentration. [file Image_7.PNG]
