# Supplementary material for: Transcriptome Analysis of Ophraella communa Male Reproductive Tract in Indirect Response to Elevated CO2 and Heat Wave
Source: Front Physiol. 2020 May 5;11:417. doi: 10.3389/fphys.2020.00417 (PMC7215069; doi:10.3389/fphys.2020.00417)
Supplement: TABLE S1 — Primers used in qRT-PCR. [file Table_1.docx]

**Table S1.** Primers used in qRT-PCR

| **Gene ID** | Primers(F: forward primer; R: reverse primer ) |
| --- | --- |
| Oc33781_g1 | F: CCTTCAGAACGAAAACGG  R: CCTCTGGAGTAGTACCTT |
| Oc36319_g1 | F: CTCTGAAGGACTCTTGGA  R: GTGGGATGTAAGCACTAG |
| Oc28991_g1 | F: TTACCAACGACGCCACTT  R: GCTGCCTTTGCCTTATTGAC |
| Oc40935_g1 | F: GATTGGAATGGGAGGTAC  R: GGTATTCCTGATCCGCAT |
| Oc44830_g2 | F: CACAGACCCAAATCAGTT  R: TTTTCATGGTTCCTCAGC |
| Oc45230_g1 | F: CAGTGTAGGTTGGCAGTATT  R: GGTGTGAGTTGCTGTAGAG |
| Oc41705_g2 | F: GTTTGTGCCCTCCTCTTC  R: GCCTGTTGACCCTTACTT |
| Oc37599_g1 | F: ATCCTACAGCAAGCAGAGAC  R: AATCCCGCTCCCATTTGA |
| Oc38167_g1 | F: CCTTATGTTACGGGTTGG  R: TGATAGCGTCTCCTGTTC |
| Oc43123_g1 | F: CGTGGGACTCTGGTTTAC  R: CGCTCGAACTGCAAATAC |
| RPL4(reference housekeeping gene) | F: TGTGGTAATGCTGTGGTAT  R: TCTAGCACTGCATGAACA |
