# Supplementary material for: Transcriptome Analysis of Ophraella communa Male Reproductive Tract in Indirect Response to Elevated CO2 and Heat Wave
Source: Front Physiol. 2020 May 5;11:417. doi: 10.3389/fphys.2020.00417 (PMC7215069; doi:10.3389/fphys.2020.00417)
Supplement: TABLE S2 — Summary of the output quality of sequencing data. [file Table_2.docx]

**Table S2.** Summary of the output quality of sequencing data.

| **Sample** | **Raw Reads** | | **Clean reads** | | **Clean bases** | | **Error (%)** | | **Q20 (%)** | | **Q30 (%)** | **GC (%)** |
| --- | --- | --- | --- | --- | --- | --- | --- | --- | --- | --- | --- | --- |
| TE_1A_1 | 20220671 | | 20173866 | | 3.03G | | 0.02 | | 99.19 | | 97.58 | 39.54 |
| TE_1A_2 | 20220671 | | 20173866 | | 3.03G | | 0.02 | | 97.97 | | 94.92 | 39.59 |
| TE_2A_1 | 20255797 | | 20209587 | | 3.03G | | 0.02 | | 99.18 | | 97.56 | 38.97 |
| TE_2A_2 | 20255797 | | 20209587 | | 3.03G | | 0.02 | | 98.00 | | 95.02 | 39.01 |
| TE_3A_1 | 19940994 | | 19895475 | | 2.98G | | 0.02 | | 99.15 | | 97.48 | 39.08 |
| TE_3A_2 | 19940994 | | 19895475 | | 2.98G | | 0.02 | | 98.07 | | 95.14 | 39.14 |
| TEck_1A_1 | 20280780 | | 20234089 | | 3.04G | | 0.02 | | 99.17 | | 97.53 | 39.41 |
| TEck_1A_2 | 20280780 | | 20234089 | | 3.04G | | 0.02 | | 98.08 | | 95.18 | 39.47 |
| TEck_2A_1 | 20073126 | | 20026946 | | 3G | | 0.02 | | 99.19 | | 97.57 | 38.83 |
| TEck_2A_2 | 20073126 | | 20026946 | | 3G | | 0.02 | | 97.90 | | 94.78 | 38.89 |
| TEck_3A_1 | 20144074 | | 20097163 | | 3.01G | | 0.02 | | 99.14 | | 97.47 | 38.41 |
| TEck_3A_2 | 20144074 | | 20097163 | | 3.01G | | 0.02 | | 98.08 | | 95.21 | 38.46 |
| MAG_1A_1 | | 20303240 | | 20252844 | | 3.04G | | 0.02 | | 98.89 | 96.91 | 40.82 |
| MAG_1A_2 | | 20303240 | | 20252844 | | 3.04G | | 0.02 | | 96.28 | 92.21 | 41.03 |
| MAG_2A_1 | | 20053525 | | 20006274 | | 3G | | 0.02 | | 98.86 | 96.84 | 40.49 |
| MAG_2A_2 | | 20053525 | | 20006274 | | 3G | | 0.02 | | 96.33 | 92.22 | 40.68 |
| MAG_3A_1 | | 20050019 | | 19998468 | | 3G | | 0.02 | | 98.80 | 96.71 | 40.35 |
| MAG_3A_2 | | 20050019 | | 19998468 | | 3G | | 0.02 | | 96.31 | 92.15 | 40.54 |
| MAGck_1A_1 | | 20040655 | | 19992722 | | 3G | | 0.02 | | 98.81 | 96.71 | 41.09 |
| MAGck_1A_2 | | 20040655 | | 19992722 | | 3G | | 0.02 | | 96.18 | 91.96 | 41.31 |
| MAGck_2A_1 | | 20125911 | | 20076763 | | 3.01G | | 0.02 | | 98.88 | 96.88 | 41.09 |
| MAGck_2A_2 | | 20125911 | | 20076763 | | 3.01G | | 0.02 | | 96.35 | 92.34 | 41.29 |
| MAGck_3A_1 | | 20133833 | | 20085259 | | 3.01G | | 0.02 | | 98.78 | 96.67 | 40.50 |
| MAGck_3A_2 | | 20133833 | | 20085259 | | 3.01G | | 0.02 | | 96.00 | 91.72 | 40.71 |
