# Supplementary material for: Transcriptome Analysis of Ophraella communa Male Reproductive Tract in Indirect Response to Elevated CO2 and Heat Wave
Source: Front Physiol. 2020 May 5;11:417. doi: 10.3389/fphys.2020.00417 (PMC7215069; doi:10.3389/fphys.2020.00417)
Supplement: TABLE S3 — Statistics of the final assembly and prediction of coding genes. [file Table_3.docx]

**Table S3.** Statistics of the final assembly and prediction of coding genes.

| **Database** | **Number of Unigenes** | **Percentage (%)** |
| --- | --- | --- |
| NR | 23120 | 34.4 |
| NT | 5361 | 7.97 |
| KO | 6167 | 9.17 |
| SwissProt | 14311 | 21.29 |
| PFAM | 17430 | 25.93 |
| GO | 17649 | 26.26 |
| KOG | 7210 | 10.72 |
| All Databases | 1364 | 2.02 |
| At least one Database | 28369 | 42.21 |
| Total Unigenes | 67205 | 100 |
