# Supplementary material for: Transcriptome Analysis of Ophraella communa Male Reproductive Tract in Indirect Response to Elevated CO2 and Heat Wave
Source: Front Physiol. 2020 May 5;11:417. doi: 10.3389/fphys.2020.00417 (PMC7215069; doi:10.3389/fphys.2020.00417)
Supplement: TABLE S4 — Distribution of single nucleotide polymorphisms (SNPs) in each sample. [file Table_4.docx]

**Table S4.** Distribution of single nucleotide polymorphisms (SNPs) in each sample.

| **Sample** | **Total SNPs** | **Non-coding SNPs** | **Coding SNPs** | **Synonymous** | **Non-synonymous** |
| --- | --- | --- | --- | --- | --- |
| MAG_1A | 100348 (100%) | 51217 (51.04%) | 49131 (48.96%) | 37270 (37.14%) | 11861 (11.82%) |
| MAG_2A | 96122 (100%) | 49533 (51.53%) | 46589 (48.47%) | 35442 (36.87%) | 11147 (11.60%) |
| MAG_3A | 104830 (100%) | 53941 (51.46%) | 50889 (48.54%) | 38495 (36.72%) | 12394 (11.82%) |
| MAGck_1A | 98299 (100%) | 48728 (49.57%) | 49571 (50.43%) | 37763 (38.42%) | 11808 (12.01%) |
| MAGck_2A | 92596 (100%) | 46302 (50.00%) | 46294 (50.00%) | 35395 (38.23%) | 10899 (11.77%) |
| MAGck_3A | 111715 (100%) | 57355 (51.34%) | 54360 (48.66%) | 40933 (36.64%) | 13427 (12.02%) |
| TE_1A | 123220 (100%) | 60961 (49.47%) | 62259 (50.53%) | 46529 (37.76%) | 15730 (12.77%) |
| TE_2A | 130735 (100%) | 65405 (50.03%) | 65330 (49.97%) | 48546 (37.13%) | 16784 (12.84%) |
| TE_3A | 129076 (100%) | 64462 (49.94%) | 64614 (50.06%) | 48146 (37.30%) | 16468 (12.76%) |
| TEck_1A | 126736 (100%) | 63185 (49.86%) | 63551 (50.14%) | 47210 (37.25%) | 16341 (12.89%) |
| TEck_2A | 138070 (100%) | 69828 (50.57%) | 68242 (49.43%) | 50507 (36.58%) | 17735 (12.84%) |
| TEck_3A | 138141 (100%) | 70422 (50.98%) | 67719 (49.02%) | 49930 (36.14%) | 17789 (12.88%) |
