# Supplementary material for: Transcriptome Analysis of Ophraella communa Male Reproductive Tract in Indirect Response to Elevated CO2 and Heat Wave
Source: Front Physiol. 2020 May 5;11:417. doi: 10.3389/fphys.2020.00417 (PMC7215069; doi:10.3389/fphys.2020.00417)
Supplement: TABLE S5 — Statistical table of FPKM values for all the samples. [file Table_5.docx]

**Table S5.** Statistical table of FPKM values ​​for all the samples.

| **FPKM Interval** | **MAG_1A** | **MAG_2A** | **MAG_3A** | **MAGck_1A** | **MAGck_2A** | **MAGck_3A** | **TE_1A** | **TE_2A** | **TE_3A** | **TEck_1A** | **TEck_2A** | **TEck_3A** |
| --- | --- | --- | --- | --- | --- | --- | --- | --- | --- | --- | --- | --- |
| 0.3–3.57 | 10514  (15.64%) | 10076  (14.99%) | 10185  (15.16%) | 10312  (15.34%) | 10201  (15.18%) | 10632  (15.82%) | 13174  (19.60%) | 13757  (20.47%) | 13840  (20.59%) | 14025  (20.87%) | 13728  (20.43%) | 14745  (21.94%) |
| 3.57–15 | 5007  (7.45%) | 4780  (7.11%) | 5119  (7.62%) | 5223  (7.77%) | 4599  (6.84%) | 5616  (8.36%) | 6486  (9.65%) | 6452  (9.60%) | 6735  (10.02%) | 6645  (9.89%) | 6605  (9.83%) | 7059  (10.50%) |
| 15-60 | 2400  (3.57%) | 2303  (3.43%) | 2578  (3.84%) | 2385  (3.55%) | 2148  (3.20%) | 3089  (4.60%) | 3768  (5.61%) | 4066  (6.05%) | 3883  (5.78%) | 3921  (5.83%) | 4056  (6.04%) | 4305  (6.41%) |
| >60 | 1101  (1.64%) | 1086  (1.62%) | 1186  (1.76%) | 1085  (1.61%) | 949  (1.41%) | 1235  (1.84%) | 2378  (3.54%) | 2473  (3.68%) | 2428  (3.61%) | 2387  (3.55%) | 2472  (3.68%) | 2573  (3.83%) |
| Specific(>0.3) | 921 | 716 | 850 | 761 | 743 | 1134 | 1459 | 1677 | 1811 | 1868 | 1497 | 2182 |
